# Ramadan fasting and hematological disorders: clinical considerations, risks, and management strategies

**DOI:** 10.46989/001c.150393

**Published:** 2025-12-11

**Authors:** Abdulrahman Nasiri, Ahmed Alahmadi, Manal Alshammari, Ruba Alabiri, Sara Samarkandi, Huda Alfattah, Rawan Alqahtani, Tariq Alzahrani, Hadeel Samarkandi, Abeer Habash, Eysa Alsolamy, Hamad Al Ghethber, Reem Alkharras, Ghada Makhdoum, Mostafa F Mohammed Saleh, Amr Hanbali, Riad El Fakih, Hazza Alzahrani, Ali Alahmari, Mahmoud Aljurf

**Affiliations:** 1 Imam Mohammad Ibn Saud Islamic University (IMSIU), College of medicine, Riyadh, Saudi Arabia; 2 King Faisal Specialist Hospital & Research Centre, Hematology, Riyadh, Saudi Arabia; 3 King Salman Specialist Hospital, Hail, Saudi Arabia; 4 King Khalid university medical city, Hematology, Abha, Saudi Arabia; 5 Department of Pharmacy, Security Forces Hospital, Riyadh, Saudi Arabia; 6 Saudi Food and Drug Authority, Riyadh, Saudi Arabia; 7 Department of Pharmacy, King Faisal Specialist Hospital & Research Centre, Riyadh, Saudi Arabia; 8 Department of Oncology, University of Toronto, Canada; 9 Department of Internal Medicine, Security Forces Hospital, Riyadh, Saudi Arabia; 10 Department of Hematology, Saudi German hospital, Jeddah, Saudi Arabia

**Keywords:** Ramadan fasting, hematological disorders, coagulation disorders, hematologic malignancies, myeloproliferative neoplasms, hydration, nutritional deficiencies, risk stratification, patient education, metabolic adaptations

## Abstract

Ramadan fasting induces significant physiological adaptations that can affect patients with hematological disorders. While fasting is generally safe for healthy individuals, reduced hydration and nutritional intake during the 13–18 hour fasting period can pose risks for those with anemia, coagulopathies, or malignancies.

High-risk patients – such as those on intensive chemotherapy, post stem-cell transplantation, or with severe anemia (hemoglobin <8 g/dL) – are advised not to fast to avoid complications (e.g. vaso-occlusive crises, thrombotic events, or worsening anemia). Patients with stable hematologic conditions, however, may fast safely with careful pre-Ramadan evaluation, medication timing adjustments, and strict hydration and nutrition plans.

Given the limited clinical data, an individualized risk stratification approach is essential. Shared decision-making, balancing religious aspirations with medical safety, is encouraged. Future studies are needed to establish evidence-based guidelines, but until then, thorough risk assessment, proactive monitoring, and patient education form the cornerstone of safe Ramadan fasting for individuals with hematological disorders.

## Introduction

Ramadan is the ninth month of the Islamic lunar calendar during which practicing Muslim adults fast daily from sunrise to sunset. This fast spans approximately 13–18 hours (depending on geography and season) and involves refraining from both food and drink.^1^ Such fasting induces various physiological changes as the body adapts to altered meal timing and sleep patterns. Notably, fluid restriction during daylight can lead to dehydration, and the shifts in routine can modulate metabolism and hormone rhythms .^2^ In healthy individuals, Ramadan fasting is generally well-tolerated and has even been associated with metabolic benefits (e.g. improved lipid profiles and insulin sensitivity).^3^ However, the situation is more complex for those with chronic medical conditions.^4^

Islamic teachings exempt the sick from fasting, yet many patients with stable conditions still choose to fast, prioritizing the spiritual significance of Ramadan. This raises important questions about safety and management. Hematological disorders—ranging from anemias and coagulopathies to malignancies—could be affected by fasting-related changes in diet and hydration. Even healthy people demonstrate some hematologic changes during Ramadan; for instance, one study noted a modest suppression of red blood cell production in healthy fasters,^5^ and mild, transient immune modulation has been observed.^2^ In susceptible patients, these changes might precipitate complications (e.g. vaso-occlusive crises in sickle cell disease or bleeding in hemophilia).

Nevertheless, literature specifically addressing Ramadan fasting in hematological diseases is limited, and robust clinical guidelines are lacking.^6^

This review provides a scientific overview of how Ramadan fasting influences on blood physiology impacts various hematological disorders. We emphasize evidence-based observations (where available) and clinical recommendations. Our goal is to aid clinicians and patients in making informed decisions that balance religious obligations with health considerations.

## Hematological Changes During Fasting

### I. Red Blood Cells, Hemoglobin, and Iron Metabolism

Ramadan fasting can lead to measurable but generally modest changes in erythropoiesis and red blood cell (RBC) indices. Some studies have reported a slight suppression of RBC production during Ramadan, evidenced by lower hemoglobin (Hb), RBC count, and packed cell volume by the third week of fasting. For example, a cohort of outdoor workers in Indonesia demonstrated significant mean Hb decline of about 1 g/dL by the end of Ramadan compared to non-fasting controls.^5^ Importantly, in these healthy individuals the red cell indices remained normocytic and normochromic, indicating a proportional reduction rather than frank iron-deficiency anemia. The likely causes are multifactorial (e.g. reduced overall nutrient intake and altered circadian rhythms of erythropoietin). Iron metabolism appears to be a key factor: one study in pregnant women found that with adequate iron supplementation, Ramadan fasting did not significantly change Hb or serum ferritin levels.^7,8^ In that study, Hb was maintained (even slightly increased) and iron stores showed only a minor, non-significant decrease over the month.^8^ These findings suggest that nutritional status is critical in mediating hematological responses: fasting itself does not inherently cause anemia if iron and calorie intake are sufficient.

### II. Coagulation Factors and Platelet Function

Dehydration during Ramadan can cause hemoconcentration and increased blood viscosity, which in turn may raise coagulability and thrombotic risk.^9^ Higher blood viscosity and concentrated clotting factors could predispose susceptible individuals to thrombosis. In fact, dehydration is known to elevate levels of coagulation factors while reducing fibrinolysis, shifting the hemostatic balance toward a pro-thrombotic state. A recent case report linked prolonged religious fasting (with inadequate fluid intake) to development of a deep vein thrombosis, attributing it to dehydration-induced thrombogenicity.^10^ Reassuringly, studies in healthy Ramadan observers have not demonstrated any increase in thrombotic events; some even report a decrease in certain pro-coagulant factors during the month. Nonetheless, patients with underlying thrombophilia or other prothrombotic conditions must be counseled to maintain fluid intake during non-fasting hours to mitigate any hemoconcentration risk.^9^

Platelet counts tend to remain within normal limits for most fasting individuals. Interestingly, one study found that patients with sickle cell disease (SCD) had slightly lower platelet counts during Ramadan: platelets dropped from a mean of ~271×10^3^/μL pre-Ramadan to ~234×10^3^/μL during fasting (a ~13% decrease).^11^ Although this drop was statistically significant, it remained well above the thrombocytopenia threshold (150×10^3^/μL), and its clinical significance is unclear. It hints that fasting might reduce certain stimuli for platelet production or activation (possibly through reduced inflammation or bone marrow activity), but more research is needed.^12^

Overall, for the general fasting population, the risk of thrombosis appears low. In those with predisposing conditions (e.g. thrombophilia, a high baseline hematocrit, or use of prothrombotic medications), fasting could become detrimental. Clinicians should remain vigilant for signs of thrombosis in high-risk patients during Ramadan and advise immediate medical attention if symptoms (like limb swelling, chest pain, or severe headaches) arise.

### III. Leukocytes and Immune Function

Ramadan fasting may exert mild and transient modulatory effects on the immune system.^3,9^ Studies have noted small declines in total leukocyte counts during the fasting period. In one cross-sectional study of healthy adults, total white blood cell (WBC) counts (including lymphocytes and neutrophils) fell significantly by the third week of Ramadan compared to pre-Ramadan values, though importantly all counts remained within normal physiological ranges.^9^ Concomitant with these cell count changes, levels of certain cytokines and immune mediators also fluctuated. Pro-inflammatory cytokines such as interleukin-1β, IL-6, and TNF-α tend to decrease during Ramadan fasting, reflecting a reduction in systemic inflammation. Similarly, acute phase reactants like C-reactive protein often show stable or reduced levels during Ramadan in the absence of other illness. These changes suggest that fasting induces an anti-inflammatory effect, which has been proposed as one mechanism behind some health benefits of intermittent fasting.^13^

Changes in humoral immunity have also been noted: for instance, immunoglobulin G and A levels have been observed to drop modestly during Ramadan though, as with cell counts, they usually stay within normal limits, and typically return to baseline by the end of the fasting month.

In summary, Ramadan fasting in healthy or stable individuals causes minor immunological changes that are usually transient. This immune modulation could even be beneficial by temporarily reducing pro-inflammatory activity, although patients with immunocompromise should still exercise caution.

**Table 1** summarizes the key hematological changes observed during Ramadan fasting.

**Table 1.** Impact of Fasting on Hematological Parameters (with clinical concern levels)

**Parameter**	**Effect of Fasting**	**Clinical Implications**	**Concern Level**
RBC Count	Mild reduction	Minimal impact unless significant pre-existing anemia. .	Low
Hemoglobin	Slight decrease	Monitor anemic patients closely for any symptomatic drop. .	Moderate
Coagulation Factors	Increased viscosity with dehydration	Elevated thrombosis risk in predisposed patients (ensure hydration).	Moderate
Leukocyte Count	Mild transient suppression	Usually benign, but may affect severely immunocompromised patients.	Low
Platelet Count	Variable (e.g. may decrease slightly in SCD patients)	Unclear significance; remains in normal range in reported studies.	Low

## Impact on Specific Hematological Disorders

### I. Nutritional Anemia

Patients with nutritional anemias (iron, B-12, or folate deficiency) may experience a worsening of anemia during Ramadan if their nutrient intake is inadequate. Reduced consumption of iron-rich foods or malabsorption (from altered meal timing or gastritis) can lead to lower Hb levels over the month. As noted, even healthy fasting individuals showed a small Hb drop (~1 g/dL) by the end of Ramadan, which was reversible after resuming normal diet. In anemic patients, a similar drop could be more clinically significant.^5^

It is therefore important to optimize nutrition in the weeks leading up to and during Ramadan. Ensuring adequate iron, vitamin B-12, folate, and overall caloric intake between sunset (Iftar) and dawn (Suhur) is essential. Adequate hydration is also crucial, as dehydration-related fatigue can compound anemia symptoms such as dizziness or weakness.^14^

From a clinical standpoint, most patients with mild to moderate anemia (e.g. Hb ~ 10–12 g/dL) who are asymptomatic can fast with close monitoring. These patients should ideally receive pre-Ramadan optimization (e.g. iron supplementation or vitamin replacement as needed) to reduce the risk of a further Hb drop during fasting. In contrast, patients with severe anemia (e.g. Hb <8 g/dL or symptomatic anemia causing fatigue, dyspnea, or cardiac stress) are generally considered high-risk if they attempt to fast. Their reduced oxygen-carrying capacity means any further Hb decline could precipitate angina, heart failure, or syncope. Such patients should be strongly advised against fasting until their anemia is corrected. Instead, they qualify for religious exemption from fasting due to illness. In all cases, close follow-up is important – for example, checking Hb mid-Ramadan for a patient with moderate anemia can guide if they should continue fasting or if interventions are needed.

### II. Hemoglobinopathies and Hemolytic Anemias

**Sickle cell disease (SCD):** Stress and dehydration are well-known triggers for vaso-occlusive crises in SCD. The concern is that daytime fluid restriction and potential lack of sleep or food could precipitate pain crises or hemolytic episodes. However, emerging data suggest that controlled intermittent fasting may not substantially increase crisis rates in stable SCD patients. A recent study from Qatar examined 52 adults with SCD who fasted during Ramadan and compared their vaso-occlusive crisis frequency in the month before, during, and after Ramadan. The study found no significant increase in severe pain crises or acute hemolytic events during Ramadan fasting compared to the non-fasting periods before and after.^11^ In fact, fasting had no obvious detrimental effect on SCD clinical outcomes in this cohort. Some laboratory changes were observed – for instance, a significant reduction in platelet count and reticulocyte count during the fasting period (as noted earlier) – but these did not translate into clinical complications. Similarly, a small case series and patient survey did not show any increase in pain crises during Ramadan among SCD patients who maintained good hydration and avoided excessive physical stress.^15^ These findings are encouraging and suggest that many patients with SCD can fast safely if they are otherwise stable and follow precautions.

Another study from Senegal focused on individuals with sickle cell trait (carriers) fasting in hot weather. It reported a significant increase in blood viscosity by evening (post-fast) in those with the sickle gene, compared to controls. The authors recommended aggressive hydration during non-fasting hours to limit this hyperviscosity risk. For SCD patients, especially those with the sickle trait or milder forms, similar advice holds: maintain adequate hydration, avoid extreme heat and strenuous activity during the day, and continue any routine medications (like hydroxyurea) on schedule.^16^

Individualization is key – patients with severe SCD phenotypes (frequent crises, organ damage, or high transfusion requirements) should be counseled that fasting is high-risk and likely not advisable. Those with milder disease who insist on fasting should have a trial period under observation and must be prepared to break the fast at the first sign of complications.

**Hypothetical Case (Sickle Cell Disease)**

A 24-year-old man with homozygous SCD (hemoglobin SS) has had two pain crises in the last year (none in the past 8 months) and is on hydroxyurea. He wishes to fast during Ramadan. His physician performs a pre-Ramadan check: Hb 9.5 g/dL, creatinine 1.0 mg/dL, and counsels him on strict nighttime hydration (at least 3 liters of water between Iftar and Suhur) and avoiding daytime exertion. The patient agrees to wake up for Suhur to drink fluids and takes his hydroxyurea in the evening. During Ramadan, he experiences mild fatigue and one episode of early pain in his back on Day 10, which resolves with hydration and analgesics without breaking the fast. He completes the month without a full vaso-occlusive crisis. This case illustrates a moderate-risk SCD patient successfully fasting with careful planning. In contrast, a 30-year-old woman with SCD who had monthly crises and baseline Hb 7 g/dL was advised not to fast; when she attempted to fast against advice, she developed severe pain and had to break her fast in the first week.

After Ramadan, follow-up for SCD patients is advisable to check for “silent” complications. For example, the Qatar study noted a small but significant rise in serum creatinine in SCD patients who fasted, suggesting some may develop dehydration-related renal stress. A post-Ramadan evaluation of kidney function and blood counts can ensure any adverse changes are addressed promptly.^15^

**Thalassemia:** Patients with thalassemia major or intermedia have chronic anemia that may raise concern during fasting. However, limited data suggest that the severity of anemia in thalassemia does not necessarily worsen with Ramadan fasting.

In one report, thalassemia patients who fasted did not show a significant drop in Hb compared to their baseline. Another study developing a thalassemia severity scoring system indirectly supports that as long as patients are transfused or managed appropriately, short-term fasting may not affect anemia severity. Nonetheless, logistical issues are important: transfusion-dependent β-thalassemia major patients cannot receive blood transfusions during daylight without breaking the fast (since transfusions are considered nourishing/prolonging life). If such a patient insists on fasting, arrangements must be made for transfusions after sunset, which can be practically challenging. In general, patients with thalassemia trait or mild forms (who are essentially asymptomatic with mild anemia) are at a low risk and usually can fast without problems. Those with transfusion-dependent thalassemia should be counseled that they qualify for exemption, but if they choose to fast, close coordination for their transfusions and chelation therapy is required.^17,18^

In summary, patients with anemia can often fast safely if their condition is stable and if they adhere to dietary recommendations, but those with severe or uncontrolled anemia should use the religious allowance to abstain from fasting for health reasons. Regular monitoring of Hb and symptoms throughout Ramadan is prudent in anemic patients who do fast.

### III. Coagulation Disorders (Hemophilia, von Willebrand Disease, Thrombocytopenia)

The act of fasting itself does not inherently worsen bleeding tendencies, since it does not directly affect clotting factor levels over such a short term. Moreover, administering clotting factor concentrates or other necessary medications by intravenous or subcutaneous routes does **not** invalidate the fast (these are considered non-nutritive injections). However, some patients mistakenly believe that any injection breaks the fast.^19^ Healthcare providers should clarify this and encourage patients to not skip prophylaxis.

For example, a patient with severe hemophilia A on regular factor VIII infusions should adhere to their infusion schedule. If an infusion is due during daylight, virtually all Islamic authorities allow it as a medical necessity. If patients are uncomfortable, the timing can be adjusted (e.g., given just before dawn or after sunset) in consultation with their medical team.

The literature on Ramadan in hemophilia is limited, largely confined to expert opinion. Generally, a well-controlled hemophilia patient (with established prophylaxis and infrequent breakthrough bleeding) can fast with low risk. They should take care to avoid trauma or heavy physical activity during the fasting day to minimize bleeding risk.

Patients with vWD or other bleeding disorders should follow similar guidance. Women with heavy menstrual bleeding might choose to use hormonal therapy to delay menses during Ramadan, which is a separate consideration but relevant for vWD patients to prevent prolonged fasting while bleeding.

**Hypothetical Case (Hemophilia)**

A 17-year-old male with severe hemophilia B (FIX <1%) is on prophylactic factor IX infusions on Monday and Thursday mornings. Ramadan is approaching, and he worries that infusing factor in the morning will invalidate his fast. His hematologist explains that medicinal injections do not break the fast and provides a letter from an Islamic authority confirming this. They agree to shift the infusion timing to just before Suhur (pre-dawn) on those days so that the patient is more comfortable. The patient fasts through Ramadan, continues his prophylaxis at the adjusted times, and experiences no bleeding episodes. This case underscores the importance of patient education and slight scheduling tweaks to ensure treatment continuity during fasting.

Patients with **severe thrombocytopenia** (platelet counts well below 50×10^3^/μL) should be careful if fasting, not because fasting will lower platelets further, but because any bleeding complication (e.g. gastrointestinal bleed or trauma) during the day cannot be managed with oral intake until they break the fast.^20^ Most such patients would be considered moderate to high risk and could be exempted depending on clinical stability. In stable chronic ITP (idiopathic thrombocytopenic purpura) with platelet counts in a safe range (>30–50k) and rare bleeding, fasting is not contraindicated. In poorly controlled ITP with active bleeding, fasting should be avoided.^21^

### IV. Thrombophilic Conditions and Thrombotic Risk

Patients with inherited or acquired thrombophilias and those with a history of venous thromboembolism (VTE) require special consideration. If they are not anticoagulated (for example, someone with a past provoked deep vein thrombosis who completed anticoagulation therapy, or a person with Factor V Leiden mutation but no thrombosis on prophylactic aspirin), they may face an increased risk of thrombosis during Ramadan due to dehydration and relative physical inactivity. As discussed, dehydration can concentrate clotting factors and reduce fibrinolysis, potentially tipping the balance toward thrombosis. Also, some individuals adopt a more sedentary lifestyle during Ramadan (e.g. staying up late at night and sleeping more during the day), which can increase stasis.

On the other hand, it is reassuring that population studies do not show a higher incidence of blood clots during Ramadan in countries where this has been examined. In fact, one older study noted decreases in fibrinogen and factor VII in healthy fasters, which might even reduce baseline coagulability. Thus, for a patient with a prior VTE now off treatment, our main advice is to remain well-hydrated and physically active (within reason) during non-fasting hours. Long periods of inactivity should be avoided; even light exercise or walks in the evening can improve circulation. If a patient has a particularly high-risk thrombophilia (for example, antiphospholipid syndrome with prior arterial clots, or recurrent unprovoked VTE), a discussion with their physician is needed to decide if fasting is safe or if temporary prophylactic measures are warranted. In many such cases, with appropriate precautions, fasting can be feasible. For patients with thrombophilia who are already on anticoagulation (see next section), the primary concern is ensuring they take their medication properly; being anticoagulated significantly lowers the risk of thrombosis, so as long as they are adherent and well-controlled, they can often fast.^9^

### V. Anticoagulation Therapy (Warfarin and Direct Oral Anticoagulants)

Patients on anticoagulants require careful management during Ramadan because changes in meal timing and diet can affect drug levels and coagulation parameters. Warfarin, in particular, is sensitive to dietary vitamin K intake. Patients may unintentionally alter their consumption of vitamin K-rich foods (like green leafy vegetables) when the eating pattern changes, potentially impacting International Normalized Ratio (INR) control. There have been conflicting reports on how Ramadan fasting influences warfarin therapy. A small prospective study noted a slight, statistically significant INR increase (mean 0.23) during Ramadan, with more frequent supra-therapeutic levels (30% versus 11%, P=0.027); however, no bleeding complications occurred.^22^ Conversely, a larger retrospective study reported stable INR values during Ramadan fasting, suggesting minimal disruption in medically stable patients.^23^

For anticoagulated patients, one strategy sometimes considered is switching warfarin to a direct oral anticoagulant (DOAC) during Ramadan for convenience, since DOACs (like apixaban or rivaroxaban) have fixed dosing and no dietary interactions. DOACs may improve adherence because they do not require frequent lab monitoring and are given once or twice daily. If a patient is already well-managed on a DOAC, they should be advised to take once-daily DOACs (e.g. rivaroxaban 20 mg daily) at Iftar, or if twice-daily (e.g. apixaban, dabigatran) to take them at Iftar and Suhur (~12 hours apart) to maintain consistent anticoagulation. Missed doses of DOACs could rapidly reduce anticoagulation, so adherence is key. A multicenter survey in 2021 found that many patients on oral anticoagulants made their own adjustments during Ramadan (such as altering dose timing around fasting), underscoring the importance of clear instructions from healthcare providers. Overall, with proper monitoring and patient cooperation, anticoagulated patients have been shown to fast without major issues.^22–25^

Patients on antiplatelet therapy like aspirin or clopidogrel generally do not require adjustments during Ramadan aside from taking pills with food at iftar to reduce gastric irritation. Low-dose aspirin has minimal impact from fasting aside from dehydration potentially slightly increasing its concentration, which is usually not clinically significant^26^

### VI. Multiple Myeloma and Thrombosis Risk in Ramadan (Lenalidomide Therapy)

Multiple myeloma patients, particularly those on maintenance therapy with immunomodulatory drugs like lenalidomide, present a special scenario during Ramadan. Lenalidomide (and its predecessor thalidomide) is well known to increase the risk of thrombosis, especially when combined with high-dose corticosteroids or chemotherapy. Reported rates of VTE in myeloma patients on lenalidomide and dexamethasone have ranged up to 20–26% in some studies. Even lenalidomide monotherapy carries a baseline VTE risk of around 3–4%.^27^ As a result, standard practice (endorsed by the International Myeloma Working Group and others) is to give thromboprophylaxis to most myeloma patients on lenalidomide-based regimens. This typically means a daily aspirin for lower-risk patients or low-molecular-weight heparin for higher-risk patients, continued throughout therapy.^28^ Such patients should be considered at least as a moderate risk for fasting.

If the patient is otherwise in remission and doing well on maintenance lenalidomide (on only aspirin prophylaxis and no active disease symptoms), they may fast with caution – ensuring excellent hydration and doing leg exercises to maintain circulation. If the patient has additional risk factors (prior history of thrombosis, obesity, or is on concurrent corticosteroids), that might bump them into a high-risk category, wherein fasting is not recommended due to cumulative risk.^27^

### VII. Myeloproliferative Disorders (Polycythemia Vera, Essential Thrombocythemia)

Myeloproliferative neoplasms such as polycythemia vera (PV) and essential thrombocythemia (ET) pose a distinctive challenge during Ramadan because these conditions are characterized by increased blood counts and a prothrombotic state even under normal circumstances. In PV, the hematocrit (Hct) is pathologically elevated due to excessive red cell production (often accompanied by elevated white cells and platelets). Keeping the Hct below ~ 45% is critical to reduce thrombosis risk.^29^ An ET patient can have platelet counts in the hundreds of thousands (often >1,000×10^3^/μL), which can cause clotting or paradoxical bleeding when extremely high. The concern is that dehydration during fasting will further concentrate the blood. Even a mild increase in Hct or platelets from fluid loss can increase viscosity and potentially trigger thrombosis or microvascular events.

Scheduling a prophylactic phlebotomy for PV patients right before Ramadan (or mid-Ramadan if needed) can help debulk their red cell mass and mitigate risk during the fasting period. ET patients might benefit from adjusting cytoreductive therapy timing (e.g. taking hydroxyurea after iftar) to ensure consistent blood levels.

The British Islamic Medical Association’s 2023 Ramadan guidance stratified uncontrolled clotting disorders as high risk for fasting. A poorly controlled PV (high Hct, frequent phlebotomy needs) effectively behaves like an intrinsic thrombophilia, and such patients should likely be advised against fasting – especially if they cannot reliably maintain hydration. On the other hand, a well-controlled ET patient (platelets in safer range, perhaps on aspirin or hydroxyurea) or a PV patient who just underwent phlebotomy and is on stable therapy might be considered moderate risk. These patients could attempt fasting if they are very keen to, but only with strong precautions and close follow-up. Any sign of thrombotic complications should prompt termination of fasting.^6^

### VIII. Chronic Leukemias on Oral Therapies

In chronic myeloid leukemia (CML) or chronic lymphocytic leukemia (CLL) patients who are in remission or on stable oral targeted therapy, fasting might be feasible.^30^ CML patients on tyrosine kinase inhibitors (TKIs) such as imatinib, dasatinib, or nilotinib must adhere to daily dosing for disease control. One case report from Qatar described a CML patient on nilotinib (which is normally twice-daily dosing) who chose to take the medication only once daily at Iftar during Ramadan. Remarkably, this patient maintained a major molecular remission despite the dose reduction, though this is only a single case and not a general recommendation.^31^ In general, CML patients should not skip doses; instead, the doses should be spaced to fit the fasting schedule (for example, a twice-daily TKI could be taken at Iftar and Suhur, roughly 12 hours apart).

For CLL patients on newer agents (e.g., ibrutinib) which are once-daily, timing at Iftar is convenient. The main risk for well-controlled chronic leukemia patients who fast is ensuring they do not miss any doses and that they maintain adequate nutrition to avoid weight loss or immunosuppression. If they are on therapies that require hydration (e.g., some chemotherapy or IV treatments), those would need to be scheduled in the evenings, or the patient may not be able to fast on those days.^32^

**Hematologic Malignancies on Intensive Therapy (Leukemias, Lymphomas, etc.)** Patients with active hematologic malignancies (such as those undergoing induction or consolidation chemotherapy for acute leukemia, or aggressive chemotherapy for lymphoma) are generally high-risk for fasting and are strongly advised against it. These treatments impose substantial physiological stress and often come with side effects incompatible with fasting. Chemotherapy frequently causes nausea, vomiting, or mucositis, requiring patients to have frequent small meals and ample fluid intake for comfort and to prevent dehydration. Many chemo regimens also mandate high fluid volumes for renal protection (e.g. with tumor lysis risks or certain drug infusions), as well as strict scheduling of supportive medications like antiemetics and steroids. Fasting in such a scenario is impractical and unsafe. Additionally, patients on intensive therapy are immunosuppressed; any dehydration or nutritional deficit could further compromise their recovery and increase infection risk.

Most religious authorities and physicians agree that patients receiving intensive cancer treatment qualify for a medical exemption from fasting. While most patients accept this guidance, some may insist on fasting. Such cases require individualized assessment, close monitoring, and possibly a modified fasting approach, such as intermittent fasting on non-chemotherapy days, though this remains inadvisable without medical clearance.^33^

In practice, for leukemia and lymphoma patients who want to observe Ramadan, clinicians should stratify them roughly as follows: Those on active intensive treatment (e.g., induction or consolidation chemo for acute leukemia, aggressive chemo for lymphoma, post-transplant immunosuppressants) are high risk – they should not fast (their condition would exempt them religiously as well, as preservation of life and health is paramount). Those in stable remission on minimal therapy might be considered moderate risk – they can fast if their doctor approves, but with a plan in place. Those in complete remission off therapy are likely similar to any other person, aside from perhaps some residual organ dysfunction; they could be low risk if cleared by their hematologist.

An important practical aspect for patients in remission on maintenance therapies is medication timing. Some maintenance treatments (like certain oral targeted agents, or prophylactic antimicrobials in survivors) are twice daily. These can often be adjusted to iftar and Suhur dosing. The key is to ensure treatment continues as prescribed. For example, a patient on daily prednisone for graft-versus-host disease should take it with iftar (and Suhur if split doses) rather than skipping it.

**Bone Marrow Transplantation:** Patients who have recently undergone hematopoietic stem cell transplantation (especially allogeneic transplant) are another high-risk group. Early post-transplant patients are typically on multiple medications (e.g. calcineurin inhibitors like tacrolimus, antivirals, antifungals, etc.) that need precise timing and adequate hydration to prevent renal damage. They may also have mucositis or gut GVHD (graft-versus-host disease) affecting oral intake. Fasting is not recommended in this setting. Even months out from transplant, if a patient is still on significant immunosuppressive therapy or has not fully engrafted, fasting could jeopardize their health. As a rule, post-transplant patients in their first 6–12 months (or longer if they have active complications) should refrain from fasting. Each case can be considered individually in later years post-transplant, but only if they are off immunosuppression and in robust health.^6^

## Clinical Recommendations

### A. Risk Stratification for Fasting

**Figure 1.**
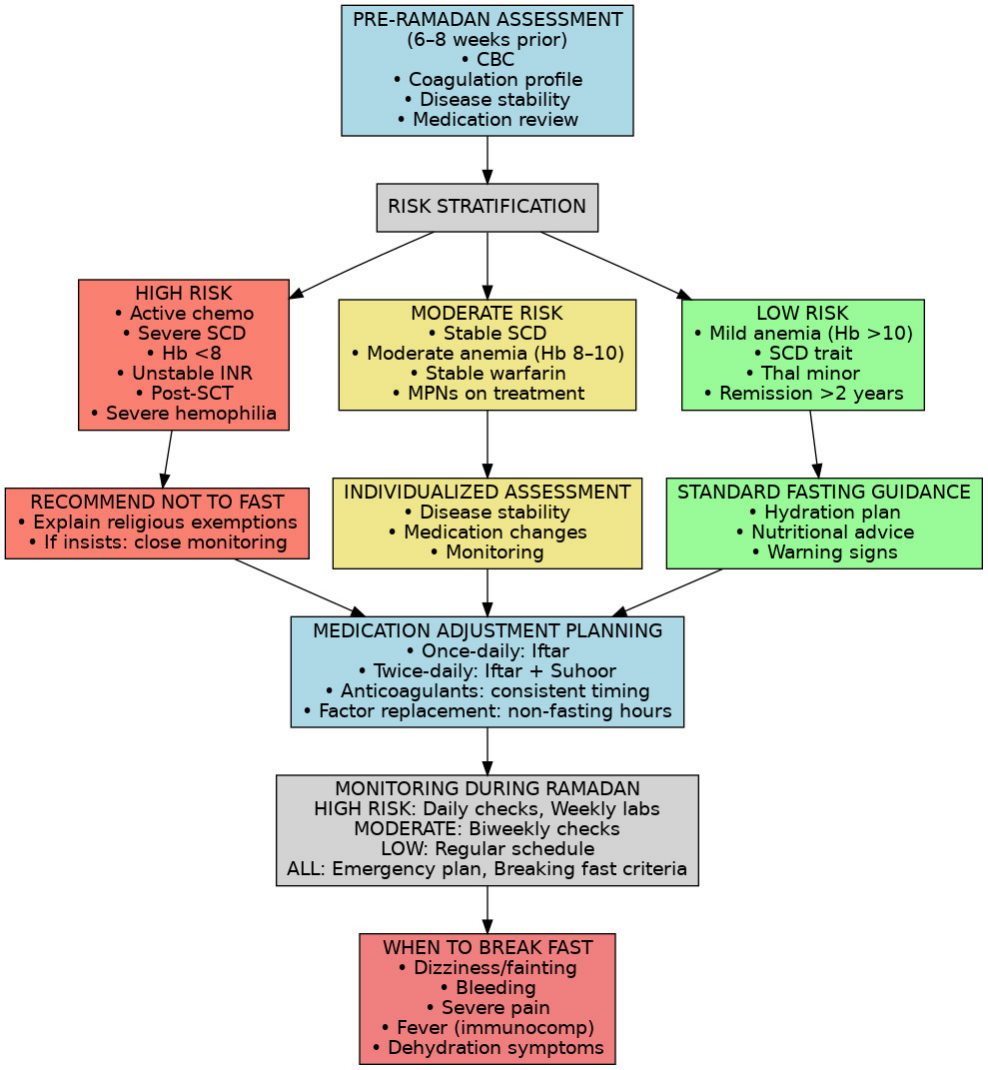
Risk stratification for fasting in hematological disorders.

Given the diversity of hematologic conditions, a risk stratification approach can help determine who can safely fast. Although no official risk stratification schema exists specifically for blood disorders, we can draw parallels from fasting guidelines in other diseases (such as diabetes). Patients should be assessed on disease activity, stability, and treatment intensity, and then categorized roughly as high, moderate, or low risk with respect to Ramadan fasting (Figure 1):

- High Risk – Fasting Not Advised: This group includes patients with severe, active, or unstable disease where fasting is likely to cause harm. Examples include sickle cell disease with frequent crises, ongoing severe hemolytic anemia requiring transfusions, any hematologic malignancy on active chemotherapy, or uncontrolled thrombophilia with recent thrombosis. These patients should be strongly advised not to fast, and reassuring them of the religious permissibility of not fasting in illness is important. “High-risk” patients are medically exempt from fasting in Islam ^29^.
- Moderate Risk – Case-by-Case Decision: Patients with stable chronic conditions or those on maintenance therapy fall here. Examples are moderate anemia that is well controlled, stable warfarin anticoagulation, well-managed PV or ET (with recent therapy and controlled counts), or leukemia patients in remission on oral targeted therapy. For these individuals, a personalized assessment is required. They may fast if their physician deems it reasonably safe, but only with a clear monitoring plan and patient education on warning signs. The decision should weigh how stable the condition is, and patients must agree to stop fasting if any complication arises.
- Low Risk – Fasting Likely Safe: Patients with mild, well-controlled disorders can usually fast without incident. Examples include iron-deficiency anemia that has been treated, carriers of hemoglobinopathies (e.g. sickle cell trait or thalassemia minor with no symptoms), or those in long-term remission from a malignancy off therapy. These individuals typically tolerate fasting well, provided they follow basic healthy practices. They should still be educated on hydration and to report any unusual symptoms, but generally they can be cleared to fast.

Clinical judgment and individual patient factors remain paramount, regardless of these categories. Within a given category, patients may have personal considerations that sway the decision (for example, a “moderate risk” patient on warfarin who has no support at home to help monitor them might be handled more cautiously).

It is essential for clinicians to involve patients in the decision-making process. A candid discussion should cover the medical risks, the religious accommodations (e.g. fidyah – feeding the poor as expiation – in place of fasting), and the patient’s personal priorities. If both parties agree to a trial of fasting, a plan should be documented, and the patient should understand under what circumstances the fast should be broken for safety. Compassion and cultural sensitivity are key – patients often greatly value the spiritual aspect of Ramadan, so even if we advise against fasting, it should be done in an empathetic manner with reinforcement that their intention is appreciated and that their health is the priority.

### B. Dietary and Hydration Strategies

Optimal diet and hydration are critical for patients with hematologic conditions who choose to fast. Adequate hydration during non-fasting hours helps prevent many potential complications (from vaso-occlusive pain to thrombosis). Patients should aim for roughly 2–3 liters of water (or caffeine-free fluids) between iftar and Suhur, distributed evenly throughout the evening and early morning ^30^.

Nutritional intake should prioritize nutrient-rich foods. Meals at Iftar should include proteins to support bone marrow function and fruits and vegetables high in vitamins like folate and vitamin C. Suhur provides critical nutritional support and should not be skipped. Anemic patients should combine iron-rich foods with vitamin C to enhance iron absorption. Immunocompromised patients, such as those with neutropenia or undergoing chemotherapy, should consume well-cooked foods and avoid raw or unpasteurized items to minimize infection risks.^34^

Patients on warfarin therapy must maintain consistent vitamin K intake, avoiding significant dietary changes during Ramadan. If dietary adjustments occur, clinicians should be informed to allow timely INR checks and warfarin dose adjustments.

Table 2 summarizes general hydration and dietary recommendations, which apply not just to healthy fasters but especially to patients with hematologic disorders who fast.

**Table 2.** Hydration and Dietary Recommendations

**Category**	**Recommendation**
Hydration	Consume at least 8 glasses of water between Iftar and Suhur, avoid caffeine and diuretics.
Iron-rich Foods	Red meat, legumes, dark leafy greens; pair with vitamin C to enhance absorption.
Meal Planning	Suhur should include proteins and complex carbs; avoid skipping it.
Anticoagulation Diet	Maintain consistent vitamin K intake to stabilize International Normalized Ratio (INR) levels.

### C. Medication Adjustments

Aligning medication schedules with the fasting routine is one of the most crucial aspects of managing patients during Ramadan. Many hematologic medications (pills or injections) can be timed during the non-fasting interval without issue. For example, if a patient normally takes an immunosuppressant thrice daily, the physician might see if a long-acting formulation exists or if a twice-daily regimen could suffice for the month.^6^ Splitting doses between Iftar and Suhur is a common strategy.

Key medication adjustments and dosing strategies are summarized in **Table 3**.

**Table 3.** Medication Adjustment Guidelines and Dosing Strategies During Ramadan

** Medication Class**	**Standard Dosing**	**Ramadan Adjustment Options**	**Monitoring Parameters**
**Anticoagulants**			
Warfarin	Once daily	Take at Iftar (evening meal)	INR weekly during Ramadan
Direct Oral Anticoagulants (once daily)	Once daily	Take at Iftar	Signs of bleeding or thrombosis
Direct Oral Anticoagulants (twice daily)	Every 12 hours	Take at Iftar and Suhur	Signs of bleeding or thrombosis
**Cytoreductive Therapy**			
Hydroxyurea	Once daily	Take at Iftar	CBC weekly or biweekly
Interferon	1-3 times weekly	Schedule on non-fasting days or after Iftar	CBC, liver function
Tyrosine Kinase Inhibitors (once daily)	Once daily	Take at Iftar	Disease-specific monitoring
Tyrosine Kinase Inhibitors (twice daily)	Every 12 hours	Take at Iftar and Suhur	Disease-specific monitoring
**Supportive Medications**			
Iron Supplements	1-3 times daily	Take at Iftar (single dose) or divide between Iftar and Suhur	Hemoglobin, ferritin
Folic Acid	Once daily	Take at Iftar	Complete Blood Count (CBC)
Vitamin B12	Once daily or weekly	Take at Iftar or schedule injections during non-fasting hours	CBC, B12 levels
**Immunosuppressants**			
Cyclosporine	Twice daily	Take at Iftar and Suhur	Drug levels, renal function
Tacrolimus	Twice daily	Take at Iftar and Suhur	Drug levels, renal function
Sirolimus	Once daily	Take at Iftar	Drug levels, CBC
**Chelation Therapy**			
Deferasirox	Once daily	Take at Iftar	Renal function, ferritin
Deferiprone	Three times daily	Not suitable for fasting unless dose adjustment approved by specialist	CBC (weekly), ferritin
Deferoxamine	Subcutaneous infusion	Schedule during non-fasting hours (overnight)	Renal function, ferritin, vision and hearing

They should be reminded that maintaining their treatment schedule is vital – missing medication could destabilize their disease (for example, skipping multiple doses of a TKI in CML can risk loss of molecular response). Using alarms and involving family members can help ensure timely medication intake.

### D. Pre-Ramadan Planning and Patient Education

A thorough medical evaluation before Ramadan is strongly recommended for patients with significant hematological conditions. Ideally, in the 1–2 months prior, the physician should assess disease stability and perform any necessary interventions (e.g. an anemic patient gets iron infusions or a blood transfusion if needed, a PV patient gets a phlebotomy, a hemophilia patient ensures factor supply, a warfarin patient checks INR). Lab tests such as hemoglobin level, platelet count, or INR should be optimized. This visit is also the time to devise the fasting plan or to advise the patient to abstain if they are high risk. Providing written instructions can be very helpful – for example, a schedule of when to take each medication, signs and symptoms that require breaking the fast, and general tips. Some experts also encourage having the patient sign an agreement or acknowledgement of the plan, especially if they insist on fasting against medical advice, to document the counseling provided.^19^

Medication adjustments require proactive planning, as unplanned changes carry risks. Regular monitoring during Ramadan helps detect issues early. Patients and healthcare providers should adopt a structured approach: plan carefully, monitor closely, and intervene promptly to ensure safe and effective fasting.

Patients attempting Ramadan fasting must be educated on symptoms that require immediate termination of fasting for medical reasons. These include severe dizziness, near-fainting episodes (suggestive hypotension or anemia), chest pain, significant bleeding, or symptoms consistent with a sickle cell crisis (e.g., severe pain). Patients should promptly break their fast if these occur. Clinicians should emphasize that Islamic teachings explicitly permit breaking the fast due to illness, prioritizing health and safety above fasting obligations.^4^ Patients should be clearly reminded that Islamic teachings prioritize health – the Quran explicitly permits breaking the fast due to illness or harm. They should feel no guilt in doing so if necessary, as it is religiously sanctioned. In fact, emphasizing this point can alleviate patients’ anxiety; many patients are relieved to hear that their faith allows flexibility in such scenarios. Involving an Imam or chaplain to reiterate this can reinforce the message and help patients accept medical advice.^35^

### E. Psychosocial and Spiritual Considerations

Finally, it is important to address the psychological aspect: for many patients, especially those with cancer or chronic illness, participating in Ramadan fasting can have emotional and spiritual benefits. It provides a sense of normalcy and hope. Physicians should approach this subject with empathy. If a patient strongly desires to fast for spiritual well-being, we should acknowledge that and work together to make it as safe as possible. On the other hand, if fasting is too risky, framing the advice in a compassionate light – e.g., “Your intention and effort are what count, and there are other ways to partake in the blessings of Ramadan” – can help. Some cancer patients have reported that even modified fasting improved their mood and coping. While safety comes first, supporting the patient’s spiritual needs is also part of holistic care.^36,37^

Finally, it’s worth reiterating the need for more research here. The literature on fasting in cancer patients is limited, and almost nonexistent for blood cancers.^6^ Future studies or at least collected clinical experiences (perhaps via case series) would help guide us. Until then, the approach will be based on clinical judgment and guiding principles from analogous situations.

## Conclusion

Navigating Ramadan fasting with hematological disorders requires balancing medical safety and religious observance. High-risk patients—those with active disease, on intensive chemotherapy, post-transplant, or with severe anemia—should abstain from fasting, while stable patients with well-controlled conditions may fast safely with proper guidance. Success depends on individualized risk assessment, thorough pre-Ramadan evaluation, and strategic management of hydration, nutrition, and medication timing. By combining scientific rigor with cultural sensitivity, healthcare providers empower patients to make informed decisions that honor both their faith and health. Islamic law provides exemptions for medical necessity—a principle that harmoniously aligns religious wisdom with clinical care.

### Authors’ Contribution

**Conceptualization**: Abdulrahman Nasiri, Ahmed Alahmdi, Mahmoud Aljurf

**Methodology**: Abdulrahman Nasiri, Ahmed Alahmdi, Sara Samarkandi, Hazza Alzahrani

**Formal analysis and investigation**: Abdulrahman Nasiri, Ahmed Alahmdi, Sara Samarkandi, Mostafa Saleh, Manal Alshammari, Ruba Alabiri

**Writing – original draft preparation**: Abdulrahman Nasiri, Ahmed Alahmdi, Sara Samarkandi, Manal Alshammari, Ruba Alabiri

**Writing – review and editing**: Abdulrahman Nasiri, Ahmed Alahmdi, Mahmoud Aljurf, Amr Hanbali, Hazza Alzahrani, Riad El Fakih, Ali Alahmari, Eysa Alsolamy, Mostafa Saleh, Huda Alfattah, Ruba Alabiri

**Resources**: Abdulrahman Nasiri, Mahmoud Aljurf, Ahmed Alahmdi, Hazza Alzahrani

**Supervision**: Abdulrahman Nasiri, Mahmoud Aljurf, Riad El Fakih, Amr Hanbali

### Competing of Interest – COPE

No competing interests were disclosed.

### Informed Consent Statement

All authors and institutions have confirmed this manuscript for publication.

## Data Availability

All are available upon reasonable request.
